# The Potential Impact of Labor Choices on the Efficacy of Marine Conservation Strategies

**DOI:** 10.1371/journal.pone.0023722

**Published:** 2011-08-24

**Authors:** Zachary D. Hughes, Eli P. Fenichel, Leah R. Gerber

**Affiliations:** 1 School of Sustainability, Arizona State University, Tempe, Arizona, United States of America; 2 School of Life Sciences, Arizona State University, Tempe, Arizona, United States of America; Institute of Marine Research, Norway

## Abstract

Conservation of marine resources is critical to the wellbeing of human communities. Coastal artisanal fishing communities are particularly reliant on marine resources for food and for their livelihoods. Management actions aimed at marine conservation may lead to unanticipated changes in human behavior that influence the ability of conservation programs to achieve their goals. We examine how marine conservation strategies may impact labor decisions that influence both the ecosystem and human livelihoods using simulation modeling. We consider two conservation strategies in the model: direct action through fisheries regulation enforcement, and indirect action through land conservation. Our results indicate that both strategies can increase the abundance of fish, and thus contribute to the maintenance of marine resources. However, our results also show that marine fisheries enforcement may negatively impact the livelihoods of human communities. Land conservation, on the other hand, potentially enhances the livelihood of the human populations. Thus, depending on management objectives, indirect or a combination of direct and indirect conservation strategies may be effective at achieving conservation and sustainability goals. These results highlight the importance of accounting for changes in human behavior resulting from management actions in conservation and management.

## Introduction

Marine conservation and fisheries management strategies generally aim to maintain species diversity or enhance fisheries [1]. However, conserving fish stocks is not synonymous with sustaining or enhancing the wellbeing of people dependent on those fisheries. Indeed, the best unconstrained way to preserve fish stocks is not to fish[2]. Conservation implies “wise use” [3], therefore, marine conservation approaches and sustainable fisheries must consider the benefits to people [4], [5]. Thus, the conservation of fish stocks is a necessary but not sufficient condition for a sustainable fishery. As such, there are more policies that can lead to the conservation of a fish stock or its enhancement than lead to long term fisheries sustainability. Strategies that best enhance or maintain fish stocks may not satisfy all the necessary conditions for fishery sustainability. Fishery sustainability may be particularly important in developing communities with coastal artisanal fisheries that are reliant on fishery resources for livelihoods and subsistence.

Marine conservation strategies often focus on establishing and enforcing regulations to curb overfishing resulting from open-access fisheries [4]. However, making marine management decisions based solely on biology, without considering how fishing incentives change behavior, may undermine ecological, social, and economic goals [4]–[7]. Furthermore, it is important to examine how costs and benefits associated with conservation actions are distributed throughout the community. Including social and economic factors in marine conservation decision-making is critical to maintaining a flow of ecosystem services [8]–[10] and preserving livelihoods for the people most impacted by marine conservation actions [11]. Research has shown that when conservation measures preserve critical services or benefit alternative livelihood options such as tourism, they may net positive socio-economic and development benefits [12].

Management recommendations based on consideration of socio-economic feedbacks often differ from recommendations that only consider ecological or economic components of the system [13]. Rondeau and Bulte [14] demonstrated that wildlife conservation efforts can have perverse impacts on wildlife populations and human welfare if conservation programs fail to account for how changes in the management or the ecosystem alter human behavioral incentives. Leslie et al. [15] modeled how different economic sectors can respond differently to ecological perturbations influencing the stability of ecological processes. Finnoff and Tschirhart [16] demonstrated the connection between ecological conditions and market prices and showed how these interactions influence human behaviors that impact the marine environment. We contribute to this literature of linked socio-ecological systems by modeling how marine conservation strategies may affect labor decisions that influence fish stocks.

Marine conservation efforts in developing regions are often organized by international NGOs or governmental agencies with simultaneous goals of enhancing human livelihoods through socio-economic development and engaging in conservation – the protection of wild populations [1]. Accordingly, we model third party's (e.g., NGO or government agency) conservation efforts, with an eye towards the development impacts. Two conservation strategies are considered: coastal land conservation (indirect marine conservation) and improving the enforcement of fisheries regulations (direct marine conservation). These conservation strategies work to preserve fish stocks by incentivizing changes in human behavior that heavily influence fish stocks. Land conservation may indirectly affect labor choices by creating incentives for tourism producers to increase employment in the tourism industry. Land conservation also influences marine life *indirectly* by reducing pollution from terrestrial areas into the marine environment. Pollution can increase mortality and reduce recruitment for marine wildlife [17], [18]. The enforcement of fishery regulations acts *directly* to limit fishing effort by creating disincentives to fishing. Enforcement of common fisheries regulations (e.g., spatial and temporal closures and gear restrictions) increases expected costs to local fishers [19], [20] all else equal, and reduces fishing effort. In both cases, the policies affect the fish stock through providing incentives in fishing behavior.

We apply a general equilibrium framework to model a real world system, linking economic sectors with the local ecology to explore how the system may operate under alternative conservation options. Our work is novel in that it integrates conservation management, condition of the fish stock, labor allocation decisions, and wage rates. We examine how incentive structures alter human behavior in efforts to conserve marine resources. We also highlight the importance of examining ecological and socio-economic linkages in order to determine how costs and benefits act on artisanal fishing communities.

We consider two economic sectors: fishing and tourism. The profits available from fishing relative to the wages available from working in tourism provide the economic incentives that motivate fishing effort. Income (i.e., wage rate) is used as the measure of local economic wellbeing in the model. We use our model to analyze the indirect effects that marine conservation has on the local economy, how changes in the local economy affect marine conservation, and whether conservation costs are internalized by the community, externalized through the resources, or shifted to other (potentially more developed) economic trading partners (e.g., tourists). This allows us to examine how the costs and benefits for marine conservation actions are distributed in order to determine whether the local community gains or losses from conservation actions.

### System background: Loreto Bay, Baja California Sur, Mexico

Loreto's primary economic sectors include artisanal fishing and tourism [21]. Eighty percent of employment in Loreto is in the tourism and fishing, and marine resources are important to Loreto's economy through both the fishery and tourism [21]. We model the fishery resource using leopard grouper life history characteristics and population estimates to represent the state of fishery resources. The leopard grouper (*Mycteroperca rosacea*) is highly targeted in artisanal fishing [22], may increase tourism demand through enhanced diving experiences [23], and plays an important role in maintaining marine ecosystem structure [24]. Ecosystem management approaches are highly desirable in fisheries [25], but are still only emerging. Most fisheries management is still single stock management; therefore, in an effort to focus on the positive influence of alternative policies, we focus on a single dominant stock.

## Methods

**Figure 1:**
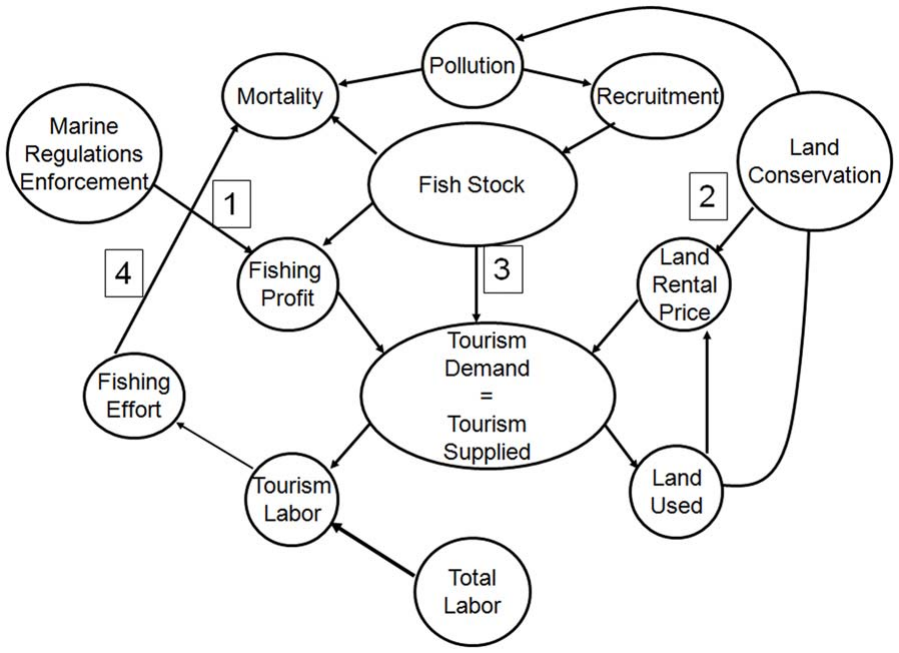
Conceptual diagram of the simulation model of primary interactions between model components. Conservation options act on the system by increasing the land rental price or increasing the cost of fishing. Pollution is driven by land use and impacts the fish stock through recruitment and mortality. Fish stock is also impacted by fishing effort through harvest. Wage rate is affected by fish stock and cost of fishing. Demand for tourism is influenced by the fish stock, while the demand, wage rate, and land rental price determine the allocation of land and tourism labor. (1) Shows where fisheries enforcement interacts with the model by increasing the cost of fishing and decreasing the profit from fishing. (2) Land conservation interacts with the model by increasing the land rental price and decreasing the amount of land used. (3) Fish stock affects the quantity of tourism demanded by increasing or decreasing the amount people are willing to pay for a unit of tourism. (4) Fishing effort alters fish mortality and controls the size of the fish stock.

We first develop a conceptual model (Fig. 1) to gain intuition about the relative effects of direct and indirect conservation actions on the local economy and labor choices. Fisheries enforcement changes how a representative individual allocates his labor, thereby altering harvest levels and impacts on the fish stock. Land conservation alters the employers' allocation of resources between labor and land, indirectly impacting harvest and fish stock. Our model describes feedbacks between the local economy and the availability of biomass available to fisheries. We then parameterize our model and simulate feedbacks over a 25 year period (Appendix S1, Table S1). We parameterize the model with data on income, harvest, employment in Loreto and biological data on the fish stocks targeted by Loreto fishers (Appendix S1, Table S2.). We establish a baseline equilibrium and then perturb the model by implementing regulatory changes to explore the impacts of our respective policy choice. This allows us to examine the dynamic feedbacks between marine wildlife and the human communities in Loreto. Our primary focus is on new ecological-economic equilibrium generated by the policy change and the implications for conservation and development.

### Model Development

We assume that land and labor are required to produce tourism, and tourism producers choose between these inputs in order to minimize production costs. Furthermore, we assume that land and labor are substitutable. For instance, the overall experience of a tourist may be improved by having more individual space, personal service, or by having more infrastructure. The substitutability may not always be direct, but producers can offer a variety of services to shift between land and labor. Fishing is always an employment option, even when there are no tourism employment opportunities; therefore, we assume full employment.

We assume that fishing has no fixed costs (i.e., boats and gear can be rented, owners of fishing gear forgo opportunity of renting out their gear to others, and there are no mooring or licensing costs in Loreto). Fishermen receive a constant market price for fish that is set by the global markets that fishers can access. Increases in tourism may effectively alter which markets that fisher's access (we'd like to thank Heather Leslie for pointing this out). Increases in tourism market may shift fish demand and increase the price of fish in two ways. First, increased tourism adds consumers, which may increase the extent of the local fresh fish market that fisher's may access. Second, the properties of the representative consumer in the local fresh fish market may change, potentially leading to greater demand and higher prices for fish. Nevertheless, the volume of local catch has no effect on the price of fish. We consider the potential effect of tourism development on fish prices in the sensitivity analysis. Marine protected areas and gear restrictions are examples of fisheries regulations that increase the costs of fishing effort, and these are types of regulations considered for Loreto Bay [23]. In our model, fisheries regulation increases the marginal costs of fishing effort, but the regulations do not affect the revenue from fish caught. Therefore, our model represents regulations such as area closures or gear restrictions such as those recently implemented in Loreto. The increased costs may be conceptualized as increased transit times, fuel costs, and loss of preferred fishing grounds (such costs would be associated with a marine reserve). We assume that there are no barriers to changing occupations, but relax this assumption in our sensitivity analysis. We also assume that there is no immigration or emigration of workers in the model. We performed sensitivity analyses by adjusting parameters individually to examine the impact of these assumptions on the qualitative nature of our results.

We model land use such that land use regulation increases the rental price of land. This simplification neglects many of the issues associated with land management and land use, such as reversibility and capital costs associated with changing land use. Inclusion of these issues may alter numerical results, however, they are unlikely alter the qualitative results.

In our model, the people purchasing units of tourism come from elsewhere, and the demand for tourism in Loreto exists in a global tourism market. Tourism demand does not depend on the disposable income of the local community. We assume a competitive tourism market in Loreto of several small producers, and accordingly model productivity. Tourists can be thought of a resource for the community of the Loreto, but consumer surplus, a measure of the net benefits to tourists from visiting Loreto, does not directly influence the wellbeing, development, and sustainability of Loreto. Furthermore, we assume that the owners of tourism producing capital are not part of the general Loreto community. Rents to land and other capital are sent out of Loreto (e.g., through bank loans). Therefore, producer surplus is not available to the citizens of Loreto. People living in Loreto only gain from tourism through wages paid for laboring in the tourism industry, which may be thought of as rent to labor. We test the assumptions about producer and consumer surplus to evaluate their impact on model results. Finally, assume that markets for tourism and labor always clear: producers always fill the full demand for tourism, and are always able to hire the cost-minimizing number workers. At each point in time, resource allocation decisions between land and labor by tourism operators are made based on the current state of the world. Tourism producers and workers do not engage in forward looking behavior, instead seek to minimize production costs or maximize income over the short term instead of seeking the optimum allocation of resources to maximize the present value of all future income.

### Model of the local labor market

Artisanal fisheries are often characterized by open access, where entry into the fishery is not limited or controlled [26]: or regulated open access management regimes where fishers to do not have secure property rights but are subject to some regulations such as gear restrictions or seasonal or area closures [27]. In both cases the resource is a common pool good. Open access and regulated open access generally imply that economic rents from fishing are dissipated and economic profits are driven to zero [27], [28]. The reason for this is that as stocks improve, additional effort enters the fishery to exploit these stocks and the system reaches a new equilibrium with zero profits [4], [27]–[29]. Homans and Wilen [27] show that even if regulations that meet biological objectives and increase yields, fisheries can still suffer from dissipation of profits if entry is not limited. The economic problem is that yield goals are met, but fishers employ excess effort, fail to operate at least cost, and drive profits to zero [4], [27], [30]. For profits to exists, the same catch would need to be achieved by fewer fishers operating at lower cost (limited entry). In our model, profits from fishing are driven to zero, but only after considering the opportunity cost of forgoing working in tourism. The opportunity costs of tourism employment prevents fishing profits from reaching zero.

In our model, workers seek to maximize net income, and we consider a representative net income maximizing individual, who divides labor between the fishing and tourism sectors. Total net income for workers is the sum of net income from fishing and net income from working in tourism. Income from working in tourism is equal to the wage rate, times labor allocated to tourism. The wage rate reflects labor market competition with fishing and is endogenously determined. In a competitive labor market, the tourism wage rate is equal to the marginal profit from fishing. Therefore, both tourism wage rate and profit from fishing are functions of fishing regulation enforcement and the fish stock.

In a competitive labor market the tourism wage rate and income from fishing equalize at each point in time, and this enables the wage rate to serve as the indicator of community wellbeing. At any instant, the representative worker takes the fish stock and enforcement as given. Though in an optimally managed fishery a larger fish stock implies greater future profits from fishing, an individual worker has no incentive to conserve the fish stock because he does not have secured rights to the stock and has no guarantee of benefiting from conservation [28], [31].

The harvest component of this model is the common Schaefer (catch-per-unit effort) harvest function [28]. This implies that the size of the fish stock effects revenue from fishing. The profitability of fishing is related to the market price of fish. There are two cost components in the fishing profit function. Both are independent of the fish stock. The first is the constant marginal cost of a unit of fishing effort, such as fuel, and boat rental (or the opportunity cost of not renting one's boat to another person). The second represents the marginal costs to the fishermen associated with fisheries regulations enforcement. It is desirable that the level of regulation is a function of the fish stock [32]. However, area closures and gear restrictions such as the regulations used in Loreto are unlikely to adapt to changes in the fish stocks, so we considered policies that are set irrespective of the fish stock.

### Tourism production

Assume that tourism employers operate in a competitive market and face a downward sloping demand function (i.e., as tourism price fails the demand level increases). The supply of tourism services is a function of tourism labor and land. We assume decreasing marginal returns to increases in either labor or land for the production of tourism services. Tourism operators are price takers for wages and land rental. We adopt the Cobb-Douglas production function as an approximation that satisfies our assumptions about the nature of tourism production, with technology parameters represent diminishing returns to scale consistent with several small producers of tourism operating in a competitive market.

Tourism producers minimize costs while meeting demand. Tourism producers take the wage rate as given due to competition with the fishing sector for labor, but choose the quantity of workers to employ. The tourism wage rate is the minimum wage that tourism operators must offer in order to make workers indifferent between fishing and working in tourism, and is equal to the marginal profit from fishing for an extra unit of time. Tourism requires space for infrastructure to operate. The area available for development is a factor in determining the cost of operating a tourism business. The land rental price is a function of land conservation. When land is conserved, the available land reduces and the land rental price increases. If land is neither conserved nor actively used for tourism, then residual land decreases the price of land rental (land is less scarce).

### Dynamic simulation methods

We use the STELLA [33] modeling environment to simulate a 25 year time horizon for three conservation scenarios: no change in conservation effort, increasing the marginal cost associated with fishing (e.g., increasing fisheries regulations), and increasing the land rental price through land conservation. Model equations are provided in Table S3. The local economy model simulates the production of tourism, allocation of workers, and the wage rate as a function of the fish stock. The dynamics come from the link between the local economy and the fish stock. The fish stock changes over time as a function of recruitment and harvest, which is a function of fishing effort and is determined by local economic conditions and biological parameters. In our simulation model, the local economy is linked to the fish stock component through the fish stock level, fishing effort, tourism demand, and pollution (Figure 1). We compare the impact of each scenario on livelihoods for workers living in Loreto and the leopard grouper stock

The fish stock is modeled using a stage structured model with two stages, juveniles and adults. A Ricker stock-recruitment function is used to determine the annual juvenile leopard grouper recruitment per adult (see Appendix S1), with recruitment from the juvenile stage to adult occurring at age four [34]. In the model, conservation strategies lead to changes in the marginal cost of fishing (fisheries enforcement) or the land rental price (land conservation). In the simulation model, we evaluate fisheries enforcement and land conservation strategies modeled by increasing the marginal cost of fishing or the land rental price by 0 to 50%. The model simulates the decentralized competitive allocation of land and labor. The supply of tourism is determined by the costs to produce tourism and yields the market clearing quantity of tourism, the point at which the tourism supplied is equal to the tourism demanded.

We consider changes to assumptions about barriers to changing occupations and capital ownerships. We investigate the effect of including costs for transitioning between occupations to test our assumption of no barriers to changing occupations because artisanal fishers are frequently the lowest income group in their communities, and fishing is generally considered the income of last resort [35]. Local ownership of capital can be important in development. Our base model assumes no local ownership of capital. We test this assumption by incorporating changes producer and consumer surplus into the welfare calculations to test the assumptions.

The simulation model is used to compare the three management strategies, the sensitivity of the results to assumptions about model structure, and the sensitivity of results to parameter values. First, we conduct sensitivity analysis with respect to the parameters, by increasing each parameter independently, over all three management scenarios. Second, we compare tourism demand that is not conditional on the state of the ecosystem with a tourism demand that shifts upward by a marine ecosystem with increased leopard grouper abundance. The upward shift in demand is motivated by Wielgus et al.'s [23] estimate of an increased willingness to pay for the chance to see more fish during tourism related diving. We calculate the change in likelihood to see grouper in the Loreto area based on Wielgus et al. [23], and then multiply this change in likelihood by the willingness to pay to see the fish [23] (also Table S2). However, this willingness to pay is an overestimation because it does not account for diminishing returns associated with viewing more wildlife [36]. In the model, the willingness to pay to see greater numbers of fish increases the choke price (the price consumers are willing to pay as available quantity of tourism goes to zero) of the demand curve thereby increasing quantity of tourism demanded at a given price.

Third, we consider how changes in the tourism demand may affect fish prices and how this may impact the results of the model. Local catch generally does not affect the price of fish; fishermen are price takers competing in a market and cannot exert control over prices. We assume that demand is derived from a mix of local and global markets. However, tourism may shift demand in the local fresh market. We consider the potential for increases in tourism to shift the demand curve for fish up (i.e., increase the choke price).

Fourth, we consider the effect of conservation actions on the local community when the fish stock is initially stable and in cases when it is declining. The decline in the fish stock is modeled as resulting from increasing the catchability coefficient, representing an increase in fishing ability (e.g., increases in fishing technology over time).

Finally, we consider the potential effects of pollution on the size of the fish stock in order to test the potential benefit to the marine environment from pollution prevention associated with the land conservation option. Pollution is modeled at various rates as proportional to the amount of land used in the production of tourism, and pollution is assumed to reduce recruitment and increase adult fish mortality.

## Results

We first examine the effects of alternative conservation strategies by exploring results derived from the labor allocation model in order to gain intuition about the system (Table 1). We then analyze simulation results from the model calibrated to Loreto for the three conservation scenarios. Parameters and data sources for the model are provided in Tables S1 and S2.

**Table 1:** Results from analytic model.

Increased Variable	Fish Stock	Wage Rate	Fishing Effort
Fisheries Enforcement	+	-	-
Land Conservation	Ambiguous	+	Ambiguous
Wage Rate	+	N/A	-
Fish Stock	N/A	+	+

Pluses indicate an increase in the stock or parameter from the column header with an increase stock or parameter in the row header, while minuses indicate a decrease.

### Summary of Generalized Model

Assuming a fixed labor supply, an increase in tourism labor implies a decrease in fishing effort. Therefore, both marine conservation strategies have the *potential* to reduce fishing effort, but through different means. Protecting land causes tourism operators to substitute labor for land; this requires a higher wage rate. A higher wage rate requires a greater return to fishing. If the return to fishing is lower, then individuals would opt to seek employment in tourism. However, the higher cost of tourism causes the quantity of tourism demanded to retract. Tourism operators require fewer workers so workers are “pushed” back to fishing. The net effect on the allocation of labor and wage rate is ambiguous and depends on the elasticity of tourism demand with respect to the price of tourism.

An external increase in fishing regulation or enforcement of regulations lowers the income for all workers throughout the economy by lowering the marginal profit of fishing. These results apply over the long term because the modeled regulations (marine protected areas, gear restrictions, etc.) result in regulated open access, and new fishers continue to have incentive enter and to compete away profits [27]. This pushes down wages since tourism operators can then substitute cheaper labor for land. Furthermore, the cheaper labor may lower the market clearing price of tourism and cause an increase in the quantity of tourism supplied. Changes in environmental quality may shift tourism demand, and changes in tourism may alter fish prices. A reduction in land use may have further conservation benefits if it reduces land-based marine pollution. Fisheries enforcement unambiguously reduces fishing effort, but the effects on wages are generally ambiguous and depend on specific relationships in the system.

**Figure 2:**
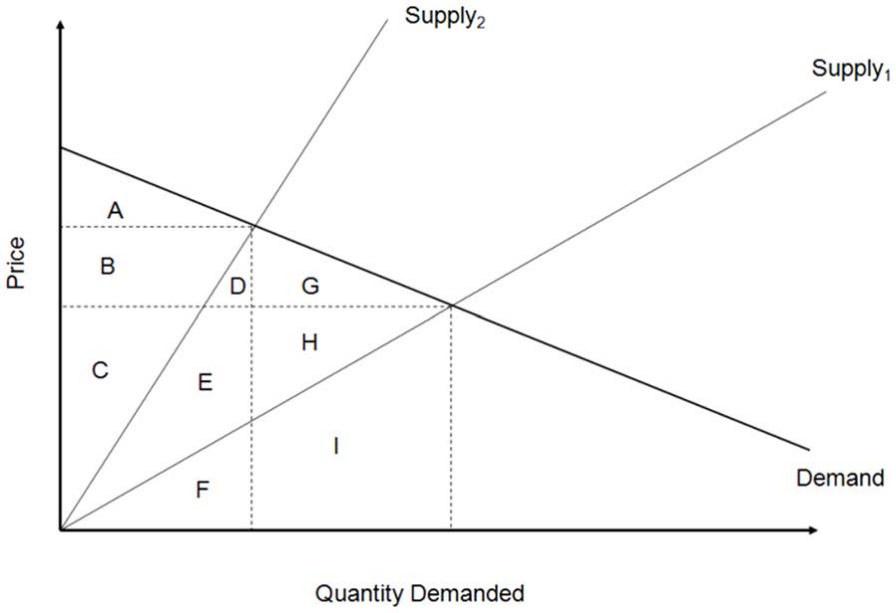
Benefit shift of conservation actions. Figure 2 illustrates how benefits are shifted with conservation action. Supply_1_ represents the initial state of our system with no conservation action, and Supply_2_ represents the new supply curve when land conservation is implemented.

Both conservation strategies have the potential to reduce fishing effort and increase fish stocks, but the cost of conservation is borne by different parties. In standard welfare analysis, attention is given to consumer and producer surplus [37]. However, in the coastal communities of many developing countries, consumer and producer surplus are exported out of the system and do not accrue to the local community. The wages paid to the local community, however, are part of what is usually not considered – wages are considered transfer payments. Figure 2 illustrates how benefits are shifted with conservation action. Supply_1_ represents the initial state of the system with no conservation action, and Supply_2_ represents the new supply curve when land conservation is implemented.

With land conservation, consumer surplus is shifted from polygon that is the sum of areas A, B, D, and G to A (Figure 2). The producer surplus changes from the polygon that is the sum of areas C, E, and H to the sum of B and C. The wages paid to the workers are initially some portion of the polygon that is the sum of areas F and I, but with land conservation wages paid to the workers are a portion of polygon D, E, F. The proportion of the polygon that is wages also changes. This indicates that with land conservation some of the costs of implementing conservation may be exported to those outside of the system and benefits transferred to local workers. Enforcement of fisheries regulations has the opposite effect and rotates the tourism supply curve down. In the case of fisheries enforcement, local workers bear the costs of conservation. Artisanal fishermen are price takers and cannot transfer the cost of conservation to consumers (this is true whether tourism affects the price of fish or not). This is a result of the elastic demand for locally caught fish. Conversely, land conversation divides conservation costs among tourism consumers (due to the downward sloping demand), tourism producers, and tourism workers (Figure 2).

Including producer or consumer surplus, or both together, does not change the qualitative results of the model. The model shows that fisheries enforcement increases consumer (tourist) surplus, while reducing producer surplus and wages (Table S4.). Land conservation decreases consumer surplus and increases producer surplus and wages (Table S4.). Thus the model demonstrates an increase in community welfare from wages and producer surplus with land conservation, and a decrease in overall community welfare as benefits are shifted to tourists with fisheries regulation enforcement. However, increasing local ownership of capital assets, which increases the amount of producer surplus that says in community, likely also enhances development, but is beyond the scope of this article.

### Simulation results

**Figure 3:**
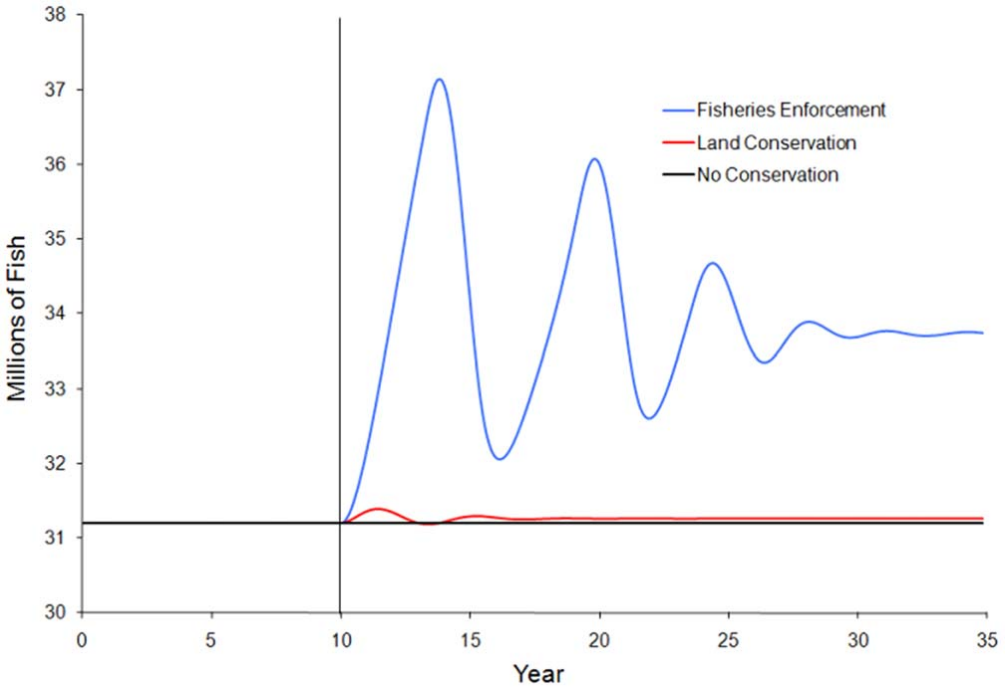
Impacts of the various marine conservation strategies on fish stocks. Conservation strategies are implemented at year 10, denoted by the vertical line. Both conservation strategies lead to an increase in fish stock in this scenario.

**Figure 4:**
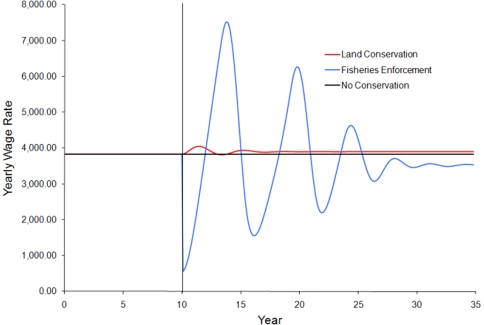
Impacts of the various marine conservation strategies on the yearly wage rate. Conservation strategies are implemented at year 10, denoted by the vertical line. Land conservation leads to an increase in the wage rate, while fisheries enforcement decreases the wage rate.

We examine results by comparing the equilibrium state prior to the implementation of conservation actions the equilibrium or near equilibrium state at the end of the simulation (T = 10 and 35 respectively). Simulation shows that both conservation strategies lead to an increase in the fish stock compared to cases with no additional conservation action (Figure 3). However, only land conservation potentially meets the socio-economic objective of enhancing livelihoods for the local population (Figure 4). Fisheries enforcement leads to a decrease in livelihoods in all scenarios. As the fish stock increases from fisheries enforcement, the wage rate begins to recover, but never returns to the level of income before regulations are enforced (Figure 4). Calculating the present value of all future income shows that fisheries enforcement leads to a 2.5–3.0% decrease in discounted present value earnings, while land conservation shows at 6.5–7.0% increase (Table 2). This shows that enforcement of fishing regulations may be the most effective way to directly conserve the fish stock, but that regulations that increase the cost of fishing likely have a negative impact on the overall livelihoods for coastal artisanal fishing communities.

**Table 2:** Present value of all future income for an individual worker with the different conservation actions.

Discount Rate	No Conservation	Fisheries Enforcement	Land Conservation
1%	$84,661	$82,193	$87,765
2.5%	$71,147	$69,215	$73,785
5%	$54,613	$53,230	$56,679
10%	$35,129	$34,139	$36,519

Results are tested over various discount rates. Fisheries enforcement leads to a decrease in the present value of all future wages of 2.5% to 3%, while land conservation leads to an increase in the present value of future wages of 6.5% to 7%.

Sensitivity analyses indicate that small changes in the parameters do not change the qualitative results of the model (Table S5.). The model is most sensitive to the technology parameters associated with the relative productivity of land and labor, β and γ. This indicates that the model is sensitive to the assumption of degree of substitutability between land and labor. More generally, it indicates that ecological outcomes may be strongly influenced by economic factors that influence how people interact with the economic-ecological system in order to generate their livelihoods.

Including fish population as a tourism demand shifter had no qualitative effect on the simulation results. This is due to the calibration of the parameters for the model. However, in general we would expect an increase in fish stocks to increase the demand for tourism. If this demand effect were sufficiently large, then it is possible that the demand increase could mitigate the wage effects from fisheries enforcement, making this a more attractive conservation option. More data on the marginal effect of increase in fish stock on tourism demand is needed to analyze the potential for such effects, but other research (e.g., [13]) suggests that environmental quality improvements have rapidly declining marginal value for tourists.

Increases in tourism demand potentially increase the price of fish. We tested several levels of tourism influence on fish prices, from no effect on the price, to the price of fish being entirely dependent on tourism. The qualitative results for the model remained unchanged, but the extent of effects were impacted. Local market impacts on fish price due to tourism create a tighter coupling between the two economic sectors. When tourism increases the demand, and hence market clearing price for fish, then enforcing fishery regulations results in less stock conservation (as a result of higher extract value), but also enables greater wage preservation as compared to a market with a globally determined price of fish. Wages are preserved because effectively fishermen are contributing indirectly to the tourism economy. The effects reverse for land conservation. When fish price is a function of tourism and the land conservation strategy is employed, then increases in land conservation yield greater stock conservation but lower wages relative to the baseline case of global market determine fish prices.

**Figure 5:**
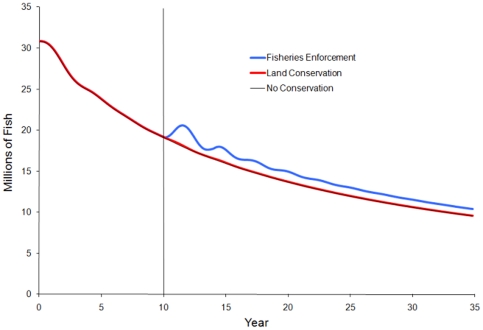
Marine conservation strategies on fish stock when the stock is declining. Fish stock is modeled as declining due to an increase in the catchability coefficient, representing improving fishing technology, with conservation strategies being implemented at 10 years, denoted by the vertical line. Fisheries enforcement slows the loss of fish stock, while land conservation has ambiguous effects on the fish stock.

**Figure 6:**
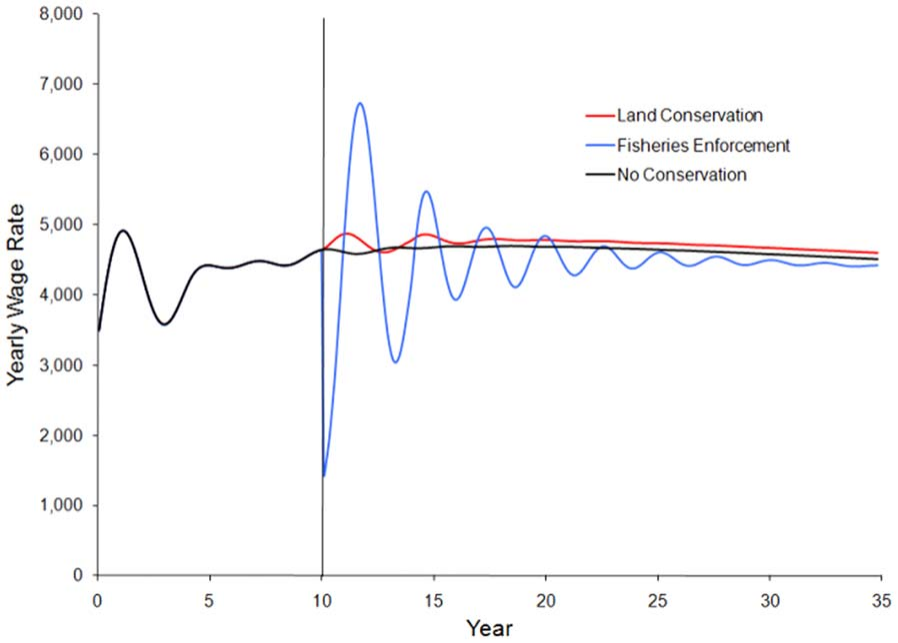
Wage rate with declining fish stock. Conservation strategies are implemented at year 10, denoted by the vertical line. Fisheries enforcement leads to a decline in the present value of all future income for workers, and also creates instability in wage rates. Land conservation leads to an increase in the wage rate. The initial instability in the model is due to the implementation of increased ability to catch fish. Due to the recruitment delay of our fish stock, it takes several years for these dynamics to stabilize.

If fish stocks decline over time as a result of increasing catchability (e.g. associated with exogenous technology or knowledge improvement), then only fisheries enforcement mitigates fish stock declines (Figure 5). Land conservation had no observable effect on the fish stock. Land conservation, however, still leads to an increase in the wage rate for the local people while fisheries enforcement lead to a decrease in the wage rate (Figure 6).

Pollution affects the model system in two ways. Higher levels of pollution lead to fish stock declines that cause wages to decline. When the size of fish stock is a factor in the demand for tourism, pollution indirectly lowers the demand for tourism, but does not alter the qualitative results. However, when pollution is decreased from land conservation, fish stock increases are limited by expanded fishing effort in response to increased profitability. Carpenter and Brock [38 indicated that an increase in fish stock might lead to increased levels of fishing; this implies that local residents may attempt to capture ecosystem service benefits generated by a cleaner environment, but these benefits are eroded by the regulated open access nature of the fishery. Moreover, there are not obvious benefits to the fish stock because of expanded fishing effort. This implies that under a regulated open access regime, there are limited benefits from mitigating pollution. Knowler [39] observes similar disincentives for the case of controlling invasive species in open access fisheries. The increased fishing effort mitigated gains to the fish stock when using the land conservation strategy with pollution effects included. However, wage rate was greater than when conservation actions were not implemented.

Modeling the inclusion of barriers to changing occupations, in the form of a cost for transitioning between working in tourism and working in fishing did not change the qualitative results of the model. Including transition costs influences the transient dynamics and leads to increased oscillations as the system moves towards equilibrium (Figure S1). The results are qualitatively similar to including open access model with sluggish entry-exit [28], [29], or time lag between ecological conditions and human response to those changes.

## Discussion

Fisheries management regulates incentive structures that influence human behaviors. In this paper, we illustrate the relative effects of alternative incentive structures in meeting socio-economic development and fisheries conservation goals. With Loreto as a real-world setting, we demonstrate how understanding how these incentives affect labor allocation decisions may play a critical role in designing conservation strategies that will be effective both in conserving marine resources and protecting the wellbeing and livelihoods of the local population. Our model provides a general framework for understanding the qualitative effects of alternative conservation strategies for marine systems. Furthermore, our approach allows explicit consideration of the tradeoffs of benefits to the marine environment and the wellbeing of human communities. Our results highlight the extent to which incentive structures in the form of conservation actions are constructed will impact who bears the costs of conservation.

Direct incentives, such as those from fisheries enforcement, may have the greatest positive impact on marine resources in the short term, but indirect incentives that work through the market may meet a wider variety of conservation and human welfare objectives. Our results indicate that land conservation (indirect conservation action) could potentially meet a wider variety of socio-economic development and conservation objectives than fisheries enforcement (direct conservation action). This may indicate that land conservation is a better balanced solution for conservation actions in terms of who bears the costs of conservation, and how the resultant benefits are distributed.

Who bears the costs of conservation depends on property rights and ownership of capital. Ownership of capital should be taken into account when measuring local social wellbeing. When residents own capital used in the production of tourism, then capital ownership can be used to indicate how changes in consumer surplus, producer surplus, or production “costs” may impact local wellbeing. If locals own tourism capital or land, then at least a portion of producer surplus goes to the local community.

Land conservation provides incentives for tourism producers to increase labor use, thereby increasing the demand for labor and necessarily increasing the wage rate, drawing people away from fishing as they seek higher income and indirectly enhancing the fish stock by reducing fishing effort. Under the land conservation option, income is increased for the local community and enhances their wellbeing while also working to conserve the marine environment. The land conservation options shifts the costs of conservation to others outside of the local system (i.e. tourists). The clear benefit to fish stocks is lost, however, in the case of unmitigated decline in the overall fish stock.

As artisanal fishing communities, such as Loreto, work to meet multiple and sometimes conflicting socio-economic and conservation objectives, our results shed light on how incentives may be structured to better meet or measure the tradeoffs of these multiple objectives. The effects of any conservation action will be filtered through the incentives that the action generates. Our model illustrates how conservation actions filter through the system to affect wage rates and ultimately affect the success of the conservation action. Further work to identify how local markets for fish products supported by tourism may extend the results of our model may increase understanding of how socio-economic development objectives and conservation objectives can work to impact the welfare of communities such as Loreto.

Fisheries enforcement imposes costs on the local community and creates incentives for tourism producers to lower the wage rate. By creating disincentives for fishing and reducing the profitability of fishing, people leave fishing to seek alternative employment, thus directly acting to reduce fishing effort and enhance fish stocks. However, this surplus of labor allows tourism producers to lower the wage rate equal to the new profitability of fishing and reduces the income for the entire community. This also indicates that land conservation may be a more desirable solution for meeting socio-economic goals for the local community because it benefits the community environment and livelihoods, while exporting some of the costs of doing so.

Our results are consistent with previous research by Allison and Ellis [6] that demonstrated that fishers' livelihoods and choices must be considered in the broader economic environment. Furthermore, the livelihoods of the entire community are linked to the abundance of the marine ecosystem. Income from non-fishing livelihoods is linked to the cost of forgoing the opportunity to fish. Therefore, when fishing becomes more profitable, the income from other forms of employment must also rise, and vice-versa. This may be a particularly useful observation for biological conservation organizations that want to work in coastal artisanal fishing communities. In these areas, it may be possible, or even necessary, to link the development agenda of poverty alleviation with the conservation agenda of preserving wildlife through different management strategies that incentivize human behavior [40]. This is different from the economic impact argument that supposes when fishing is profitable, the profit is spent in the community and the effects multiply and create more jobs.

Our model demonstrates that human well-being objectives associated with economic development and conservation objectives of preserving marine resources may be aligned with due planning, but that they are not necessarily consistent with one another. It is important to consider how policies geared towards sustainability and fisheries conservation create incentives for certain human behaviors, behaviors that ultimately determine the success or failure of such policies.

## Supporting Information

Figure S1 **Wage rate with and without barriers to transitioning.** Conservation strategies are implemented at year 10. Including a cost for transitioning between fishing and tourism shows an increase in systemic oscillation as the system moves towards equilibrium, but qualitatively similar to the results as described in the results section.
(TIFF)

Table S1 **Parameters and values used in simulation model.**
(DOCX)

Table S2 **Data and data sources for model.**
(DOCX)

Table S3 **Equations used in simulation model.**
(DOCX)

Table S4 **Results of including producer surplus, consumer surplus, and both producer and consumer surplus in addition to wages when accounting for effects on community welfare.**
(DOCX)

Table S5 **Sensitivity analysis of parameters used in simulation model.** Each parameter was increased by 5% to determine the relative impact on fish stock and the wage rate.
(DOCX)

Appendix S1. (DOCX)
