# Supplementary figures and images for: The Potential Impact of Labor Choices on the Efficacy of Marine Conservation Strategies
Source: PLoS One. 2011 Aug 24;6(8):e23722. doi: 10.1371/journal.pone.0023722 (PMC3161065; doi:10.1371/journal.pone.0023722)

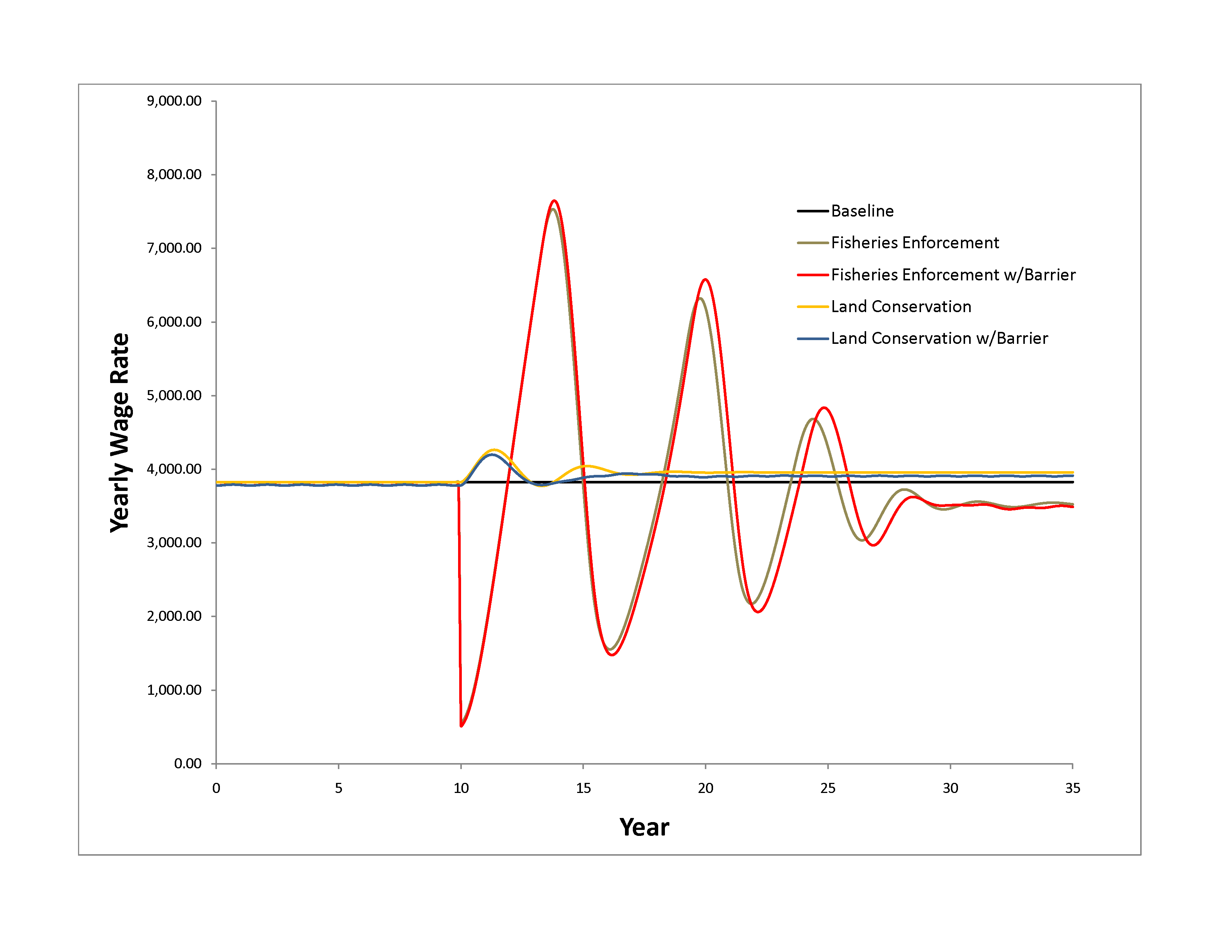

Supplement: Figure S1 — Wage rate with and without barriers to transitioning. Conservation strategies are implemented at year 10. Including a cost for transitioning between fishing and tourism shows an increase in systemic oscillation as the system moves towards equilibrium, but qualitatively similar to the results as described in the results section. (TIFF) [file pone.0023722.s001.tiff]
