# Supplementary material for: The Potential Impact of Labor Choices on the Efficacy of Marine Conservation Strategies
Source: PLoS One. 2011 Aug 24;6(8):e23722. doi: 10.1371/journal.pone.0023722 (PMC3161065; doi:10.1371/journal.pone.0023722)
Supplement: Table S1 — Parameters and values used in simulation model. (DOCX) [file pone.0023722.s002.docx]

**Table S1.** Parameters and values used in simulation model.

| Parameter | Symbol | Value |
| --- | --- | --- |
| Coefficient of production | α | 100 |
| Labor technology parameter | β | 4.67*10^-1^ |
| Land technology parameter | γ | 3.075*10^-1^ |
| Density dependent recruit mortality | κ | -4.875*10^-8^ |
| Juvenile grouper mortality | Mj | 2*10^-1^ |
| Adult grouper natural mortality | Mn | 1.8*10^-1^ |
| Low pollution | - | 1*10^-4^ * Land Used |
| High pollution | - | 1*10^-3^ * Land Used |
| Marginal Cost of Fishing | z | 3.267*10^3^ |
| Marine enforcement costs | E | 10% * z |
| Land rental price | p | 15 * Land Used |
| Land conservation costs | A | 10% * p |
| Catch coefficient | q | 7.5*10^-5^ |
