# Supplementary material for: The Potential Impact of Labor Choices on the Efficacy of Marine Conservation Strategies
Source: PLoS One. 2011 Aug 24;6(8):e23722. doi: 10.1371/journal.pone.0023722 (PMC3161065; doi:10.1371/journal.pone.0023722)
Supplement: Table S2 — Data and data sources for model. (DOCX) [file pone.0023722.s003.docx]

**Table S2.** Data and data sources for model.

| Category |  | Source |
| --- | --- | --- |
| Loreto fishers | 8750 | CONAMP-SEMARNAT, 2002 |
| Loreto tourism workers | 1250 | CONAMP-SEMARNAT, 2002 |
| Average Income | $3,830 | IMSS, 2009 |
| Adult grouper natural mortality | 0.175 | Wielgus et al., 2007 |
| Juvenile grouper natural mortality | 0.2 | Wielgus et al., 2007 |
| Grouper recruitment rate | 1.23/Adult | Wielgus et al., 2007 |
| Willingness to pay estimate | $0.60 | Wielgus et al., 2008 |
