# Supplementary material for: The Potential Impact of Labor Choices on the Efficacy of Marine Conservation Strategies
Source: PLoS One. 2011 Aug 24;6(8):e23722. doi: 10.1371/journal.pone.0023722 (PMC3161065; doi:10.1371/journal.pone.0023722)
Supplement: Table S3 — Equations used in simulation model. (DOCX) [file pone.0023722.s004.docx]

**Table S3**. Equations used in simulation model.

| Labor Market | $T=s+f$ |
| --- | --- |
| Total Net Income | *W = w(·)s + Π(f , x, r)* |
| Fishing profit | $\Pi=\left[\pi qx-(z+y(r))\right]f$ |
| Tourism wage rate, equal to marginal profit from fishing | $w=\frac{\partial\Pi(f,x,r)}{\partial f}$ |
| Wage rage, equal to marginal profit from fishing | $w\left(x,r\right)=\frac{\partial\Pi\left(f,r,x\right)}{\partial f}=\pi qx-(z+y(r))$ |
| Tourism production | $\upsilon_{s}=\alpha S^{\beta}l^{\gamma}$ |
| Fish production, Ricker Stock-Recruitment function | $R_{t}=rx_{t}e_{t}^{-\kappa x_{t}}$ |
| Stock dynamic equation | $x_{t+1}=R_{t}+x_{t}e^{-(m+qf)}$ |
