# Supplementary material for: The Potential Impact of Labor Choices on the Efficacy of Marine Conservation Strategies
Source: PLoS One. 2011 Aug 24;6(8):e23722. doi: 10.1371/journal.pone.0023722 (PMC3161065; doi:10.1371/journal.pone.0023722)
Supplement: Table S4 — Results of including producer surplus, consumer surplus, and both producer and consumer surplus in addition to wages when accounting for effects on community welfare. (DOCX) [file pone.0023722.s005.docx]

**Table S4.** Results of including producer surplus, consumer surplus, and both producer and consumer surplus in addition to wages when accounting for effects on community welfare.

| Benefit Impact (percent change from taking no action) | | |
| --- | --- | --- |
|  | Fisheries Enforcement | Land Conservation |
| Wages | -7.84% | +3.42% |
| Wages + Producer Surplus | -3.23% | +1.17% |
| Wages + Consumer Surplus | +.23% | -2.03% |
| Wages + Prod. & Cons. Surplus | +.24% | -1.63% |
