# Supplementary material for: The Potential Impact of Labor Choices on the Efficacy of Marine Conservation Strategies
Source: PLoS One. 2011 Aug 24;6(8):e23722. doi: 10.1371/journal.pone.0023722 (PMC3161065; doi:10.1371/journal.pone.0023722)
Supplement: Table S5 — Sensitivity analysis of parameters used in simulation model. Each parameter was increased by 5% to determine the relative impact on fish stock and the wage rate. (DOCX) [file pone.0023722.s006.docx]

**Table S5.** Sensitivity analysis of parameters used in simulation model. Each parameter was increased by 5% to determine the relative impact on fish stock and the wage rate.

| Baseline Values | - | 3.12*10^7^ (Fish) | 3.8*10^3^ (Dollars) |
| --- | --- | --- | --- |
| Parameter | Symbol | % Change in fish stock | % Change in wage rate |
| Coefficient of production | α | -0.68% | -6.61% |
| Labor technology parameter | β | -3.11% | -29.82% |
| Land technology parameter | γ | -1.82% | -17.42% |
| Density dependent recruit mortality | κ | -0.46% | -4.46% |
| Marginal cost of fishing | z | 4.06% | -4.05% |
| Price of fish | Π | -4.29% | 4.62% |
| Demand slope | - | -1.00% | -9.63% |
| Catch coefficient | q | -4.42% | 3.34% |
| Adult grouper natural mortality | Mn | -0.21% | -2.04% |
| Land rental price | p | 0.10% | 0.86% |
