# Supplementary material for: The Potential Impact of Labor Choices on the Efficacy of Marine Conservation Strategies
Source: PLoS One. 2011 Aug 24;6(8):e23722. doi: 10.1371/journal.pone.0023722 (PMC3161065; doi:10.1371/journal.pone.0023722)
Supplement: Appendix S1. — (DOCX) [file pone.0023722.s007.docx]

# THE POTENTIAL IMPACT OF LABOR CHOICES ON THE EFFICACY OF MARINE CONSERVATION STRATEGIES

# APPENDIX: MODEL, DATA, AND DATA SOURCES

**Simulation Model Calibration and Parameterization**

All simulation modeling was completed using STELLA (ISEE Systems Inc. 2009). Existing data for the life history characteristics of leopard grouper are used when available, and estimates based on other similar fish are used when leopard grouper data are not available (Table S2). Rates of mortality were estimated based on Wielgus et al. (2007), using the estimates of juvenile mortality (0.2) and adult mortality (0.175) from estimates of *Mycteroperca microlepis* mortality by (Heppell et al. 2006). Recruitment to the adult stage was calculated by number of juvenile recruits per adult, 3.0 (Wielgus et al. 2007), then carrying out the juvenile mortality (0.2) over the 4 years of the juvenile life stage. Density dependent mortality was solved for the value that provides an estimated unharvested fish stock of 4.0 × 10^7^ fish using a Ricker Stock Recruitment function, with a known maximum estimated recruitment: *R* = δ*xe*^-κ^*^x^* , where *δ* is the maximum recruits per adult, *x* is the stock of reproductive adult fish, and κ is the density dependence parameter.

Estimates of worker allocation came from CONAMP-SEMARNAT (2002) based on population estimates and percentages of workers in each occupation. A productivity factor, (α), of 100 was chosen based on the scale of production of tourism for Loreto. Technology parameters, ($\beta,\gamma$), were arrived at by iteratively testing the model and adjusting parameters until estimated tourism demand was met by the actual allocation of workers for Loreto (CONAMP-SEMARNAT 2002). Marginal costs of fishing, *z*, were calculated by subtracting the average worker income per year from the yearly revenue from fishing (dock price of fish * biomass) (Wielgus et al. 2007). Catch coefficient, *q*, was calculated from the harvest size and equilibrium fish stock size to match actual estimated harvest (Wielgus et al. 2007).

**Appendix References**

Conrad JM (1999) Resource Economics. New York: Cambridge University Press. 224 p.

ISEE Systems Inc. (2008) STELLA 9.1 (computer program). Lebanon, NH.

CONAMP-SEMARNAT (Comisión Nacional de Áreas Naturales Protegidas) (2002) Management program-Loreto Bay National Park. Second edition. CONAMP, Mexico City.

D’Agrosa, C, Gerber LR, Sala E, Wielgus J, Ballantyne IV F ( 2007) Navigating Uncertain Seas: Adaptive monitoring and management of Marine Protected Areas. [Online] URL:http://www.public.asu.edu/~lrgerbe/marinereserves.htm

Heppell SS, Heppell SA, Coleman FC, Koenig CC (2006) Models to compare management options for a protogynous fish. Ecological Applications 16: 238-249.

Keen EA (1991) Ownership and productivity of marine fisheries resources. Fisheries 16(4): 18-22.

Panayotou T (1982) Management concepts for small-scale fisheries: Economic and social aspects. Rome: FAO Fisheries Technical Papers No. 228. 53 p.
